# Translation and linguistic validation into Spanish of the Owner-Reported Outcome Measure “Liverpool Osteoarthritis in Dogs”

**DOI:** 10.3389/fvets.2024.1360926

**Published:** 2024-02-20

**Authors:** María Olcoz, Miguel Ángel Cabezas, Ignacio A. Gómez de Segura

**Affiliations:** ^1^Department of Animal Medicine and Surgery, University Complutense of Madrid, Madrid, Spain; ^2^Dolorvet Anestesia y Analgesia Veterinaria, Madrid, Spain

**Keywords:** chronic pain, pain assessment, osteoarthritis, questionnaire, dog, Owner-Reported Outcome Measures

## Abstract

**Introduction:**

Assessing chronic pain in dogs has been greatly favoured by the development of Owner-Reported Outcome Measures. Among them, the Liverpool Osteoarthritis in Dogs (LOAD) has been widely used for this purpose. Most of these tools have been written in English and its use by non English natives requires not only translation but also linguistic validation for use by veterinarians and owners. For its use, the LOAD has not undergone translation into Spanish and the objective was to generate a linguistically validated Spanish translation of the LOAD.

**Methods:**

Following the World Health Organisation and the International Society for Pharmacoeconomics and Outcomes Research published guidelines, the original LOAD English version underwent analysis and translation by two native linguists proficient in the target language. Both translations were then reviewed by a third native linguist to identify potential disparities and establish a cohesive translation (reconciliation). Subsequently, an independent linguist, fluent in both English and the target language, conducted the back translation. Finally, the research team compared the original and back translated versions to pinpoint and resolve any significant differences. Following the creation of the translated version, a cognitive debriefing was conducted to assess the questionnaire within the target population.

**Results:**

A total of 89 surveys were distributed to dog owners of varying ages, genders, and socioeconomic backgrounds. Although there were some suggestions and comments, and some adjustments were made, all respondents found the survey to be clear, achieving a linguistic validation of the Spanish LOAD.

## 1 Introduction

Osteoarthritis (OA) is the most commonly diagnosed joint disease in veterinary medicine, it is estimated that 20–37% of dogs aged >1 year are affected (1–3). It has a significant negative impact on the well-being and quality of life of patients, characterised by reduced mobility, alterations in activity patterns, changes in behaviour, and considerable healthcare costs (4, 5). Assessing activity and pain in chronic diseases like canine osteoarthritis is challenging because it progresses slowly, and individual effects can be relatively small (6). Additionally, associated clinical signs are more subtle, intermittent, and often have a slow onset, resulting in gradual behaviour changes (7).

For proper osteoarthritis management, it is essential that veterinarians have the appropriate tools to assess and monitor the disease progression in each patient, choose the most appropriate treatments, and study their effectiveness. Various Owner-Reported Outcome Measures (OROMs) have been developed for this purpose, measuring pain and difficulty in performing daily activities (4). These OROMs (also known as Clinical Metrology Instruments) are derived from those used in human medicine, where information is directly collected from the patient (symptoms, health-related quality of life, or functional status) (8). They include sequences of questions or items scored based on the observer's observations or experiences, typically the owner (5).

A relevant OROM among these is the Liverpool Osteoarthritis in Dogs (LOAD) scale. Initially developed to assess dogs with elbow osteoarthritis (6), it has also proven to be useful for assessment of OA in other joints (5, 9–11). LOAD consists of 23 questions, with three related to patient history, seven to lifestyle, and 13 to mobility, evaluating the impact of joint diseases on the patient's daily activity. It provides a “LOAD score” indicating the presence and severity of the patient's joint disease (Mild, 0–10; Moderate, 11–20; Severe, 21–30; Extreme, 31–52).

LOAD was originally written in English but for its global implementation in clinical practise and accessibility to all veterinarians and owners, translation into different languages is mandatory. Linguistic validation is the process by which the cultural adequacy and conceptual equivalence of translated elements are assessed to ensure that the content validity of the original element is not affected by translation (12). This reduces the risk of data invalidity resulting from incorrect translation and ensures that variations in population responses are attributable to genuine differences rather than discrepancies caused by inappropriate data collection methods (13).

LOAD has been translated and psychometrically validated to Portuguese (4) but has not been translated nor validated to Spanish. Therefore, the aim of this study was to develop a conceptually equivalent and culturally relevant version of LOAD for use in Spanish. The main hypothesis was that a valid translation into Spanish (spoken in Spain; hereafter considered as Spanish in the article) of LOAD, culturally and conceptually equivalent to the original English version, could be generated.

## 2 Materials and methods

Following authorisation from the developer (Dr. John Innes), the translation of the original version of LOAD into Spanish was carried out following the guidelines and recommendations established by the World Health Organisation (WHO), the International Society for Pharmacoeconomics and Outcomes Research (ISPOR) and Beaton et.al (12, 14–16). The selected translators for this task had previously worked on translating another OROM related to osteoarthritis in dogs. Given their experience in the subject matter, it was deemed unnecessary to provide them with detailed information or recommendations on how to carry out the translation.

Initially, two native speakers of the target language (Spanish) independently performed the direct translation (from English to Spanish). One of them was a veterinary professional with technical knowledge and familiarity with LOAD, while the other was an individual with no background in health sciences. Since the LOAD is a questionnaire directed at pet owners without technical knowledge, it was decided to include a non-veterinarian to perform one of the direct translations. Subsequently, a third Spanish linguist and a veterinary professional compared the two direct translations to identify any discrepancies to create a unified direct translation (reconciliation).

In the next step, an independent linguist proficient in native-level English and fluent in the target language drafted the back translation, i.e., translated the unified document in Spanish back to the original language (U.S. English).

Following this, the research team, along with one of the involved linguists, conducted a thorough review to examine and resolve any discrepancies between the direct translation into Spanish, the back translation into English, and the original document. This process also ensured the clarity of wording and translation concepts. If necessary, in case of significant differences or identification of comprehension issues in the target language, a new translation would be undertaken.

Once the translation process was completed, a cognitive analysis was performed to assess the questionnaire in the target population. The questionnaire employed in the study was approved by the institutional ethics committee (Ref: CE_20230511-02_SAL, 11 May 2023). Although the WHO and ISPOR recommend a minimum of five individuals to conduct this cognitive assessment up to 10 individuals per section, a total of 72 surveys were conducted with dog owners of different ages, genders, and socioeconomic characteristics to ensure representation of the target population. They were provided with a questionnaire containing a brief description of the LOAD and the objective of this study. Participants were asked to read the translated version of LOAD, indicate if each question and answer option was clearly understood, and suggest possible alternative responses (phrases or words) that they believed would facilitate comprehension of the question.

Finally, the research team evaluated all the questionnaires to determine if any modifications were necessary and, thus, to ultimately obtain a definitive translation of LOAD in Spanish.

As several modifications were made after the cognitive analysis, it was decided to repeat this analysis with a smaller number of participants (*n* = 17) to check whether the changes made posed any understanding issues.

## 3 Results

The process of translation and linguistic validation, along with cognitive analysis, allowed the development of a version of LOAD translated into Spanish that is conceptually equivalent to the original English version.

### 3.1 Translation

The direct or independent translations performed by the two native linguists were very similar, although there were 18 discrepancies between both versions. In the following step, a third native Spanish linguist compared and unified both versions to create a unified translation, selecting terms from the translation that were deemed more appropriate (considering the original version) or using different terms that were more similar to the original document. For example, in Question 1 of the Lifestyle section, “In the last week, on average, how far has your dog exercised each day?” the response options were written in miles (0–0.06 miles, 0.6–1.2 miles, etc.) in the original version. However, since distance is measured in kilometres in Spain according to the International System of Units, it was decided to convert miles to kilometres (0–1 km, 1–2 km,…), rounding to the nearest figure (0.6 miles = 1 km). Also for this question, the independent translators proposed “*En la última semana, de media, ¿cuánto ejercicio ha hecho su perro cada d*í*a*?” but the researchers ultimately decided to translate it as “*En la última semana, de promedio, ¿cuánta distancia ha recorrido su perro cada d*í*a*?” as it was considered to make more sense, given that the answers refer to the distance the dog covers during exercise, not the amount of exercise done. Annex I shows all the differences between both independent translations and the unified translation.

In the back-translation or reverse translation step, 57 discrepancies were identified compared to the original version. The research team analysed all discrepancies between the unified translation, the back-translation, and the original version. In case of persisting discrepancies, consultation with the native English-speaking linguist was done. Some changes were considered irrelevant as they were synonyms and could be used interchangeably. For example, the term “*correa*” was back-translated as “lead,” while in the original version, it was “leash.” Another example is the term “*aceptable*,” translated as “acceptable,” corresponding to the original term “fair.” In other cases, the back-translation did not exactly match the original version, but differences were very subtle and did not alter the statement's meaning. For example, in Question 3 in the Background section, the back-translation was “If you can, make a list of the medications your dog is taking…” while the original document states “If you can, please list any medications that your pet is currently receiving…” In some cases, a decision was made to make a modification based on the back-translation, such as the response option for Question 5 in the Lifestyle section “Over rough ground,” which was translated as “*Sobre terreno accidentado*” and back-translated as “Broken ground.” It was decided to change it to “*Sobre terreno irregular*” based on this difference between the back-translation and the original version, although finally changed to “*terreno accidentado*” based on the cognitive debriefing (see below). In total, out of 57 differences, only 17 terms or sentences were modified after comparing the back-translation with the original version and observing any significant differences. Annex II shows all the differences between the original version and the back-translation, as well as the modifications made to the final translated version.

### 3.2 Cognitive debriefing

Once the translation process was completed, a total of 72 dog owners were surveyed to assess the readability and understanding of the LOAD translated into Spanish. Participants were categorised by age, gender, and education level. Of these 72 individuals, 32 were men and 40 were women. The age ranges included were: ≤ 29 years (*n* = 9), 30–39 years (*n* = 15), 40–49 years (*n* = 15), 50–59 years (*n* = 23), ≥60 years (*n* = 10). The education level considered included primary school (*n* = 6), secondary school (*n* = 10), high school (*n* = 17), and postgraduate studies (*n* = 39). Overall, all participants understood without difficulty each element of the LOAD and its response options. Nevertheless, a total of 11 individuals suggested changing some sentences or words to make the reading more straightforward.

Nine participants had difficulty with Question 3: “¿*Cómo hace el ejercicio?*” (“What type of exercise is this?”) regarding the response option “*Trabajando*” (“Working”), indicating that they did not understand this concept. The research team decided to add a comment in the final translation: “*Trabajando (perro de trabajo*)” [Working (working dog)] to explain that the physical activity performed was as a working dog. Other alternatives, such as “*perro de asistencia*,” “*perro de apoyo*,” or “*perro de servicio*” were not considered, as these activities are focused on specific tasks, such as assisting people with disabilities. In addition, three respondents indicated that they did not fully understand this question, not knowing whether it referred to previous Questions 1 (“… how far has your dog exercised each day?”) and 2 (“… how many walks has your dog had each day?”) or to another specific activity. Another respondent commented that it closely resembled Question 6. “*Durante el ejercicio, ¿cómo lleva a su perro?*” (“At exercise, how is your dog handled?”) and did not understand the differences between both questions, suggesting that they should be integrated into one. It was decided to rewrite this question as “¿*Qué tipo de actividad es esta?*” (“What type of activity is this?”) because it does refer to the previous questions. Additionally, the term “*ejercicio*” (“exercise”) was replaced with “*actividad*” (“activity”) since both concepts are synonymous, and in this case, the question is related to the dog's daily activity, not just when exercising.

There was a suggestion to change Question 6 in the Lifestyle section “*Durante el ejercicio, ¿cómo lleva a su perro*?” (“At exercise, how is your dog handled?”) to “*Durante el ejercicio, ¿cómo va su perro?*” indicating a grammatical inconsistency in the Spanish translation between the question's subject (the owner) and the response options' subject (the dog). In other words, the question asks how the owner leads their dog (with a leash, without a leash, etc.), but the answers refer to how the dog goes (walks, trots, etc.). The research team confirmed this grammatical discrepancy and decided to modify the question to “*Cuando hace ejercicio, ¿cómo va su perro?*” (“When exercising, how does your dog do?”).

One participant indicated that he found the answer to question 5 “*Sobre terreno irregular*” confusing, as the other options (In the forest, in the street…) can also be irregular. The research team decided to re-translate it as “*Sobre terreno accidentado*,” to better differentiate the response options and to indicate that this is a rough and complicated terrain.

Two participants proposed that, in Questions 1 “¿*Cómo es la movilidad de su perro en general?*” (“How is your dog's mobility in general?”) and 6 “*Cuando hace ejercicio, ¿cómo de activo es su perro?*” (“At exercise, how active is your dog?”), despite correctly understanding the response options, they would change “*Pobre*” and “*Muy pobre*” to “*Mala*” and “*Muy mala*,” respectively. Further to this, another participant indicated that did not understand the terms “*Pobre*” and “*Muy pobre*,” finding them subjective. The research team decided to change the terms “*Pobre*” and “*Muy pobre*” to “*Mala*” and “*Muy mala*” both in Question 1 and Question 6, as in Spanish, “*Pobre*” and “*Muy pobre*” are usually used as adjectives to indicate something humble or scarce in economic terms, while “*Mala*” and “*Muy mala*” are adjectives used to indicate a negative value. One of these same participants also suggested that in the response options for Questions 5 “¿*Hasta qué grado su perro muestra rigidez en la extremidad afectada después de estar tumbado?*” (“To what degree does your dog show stiffness in the affected leg after a “lie down”? “) and 12 “¿*Hasta qué grado su perro muestra rigidez en la extremidad afectada después de haber estado tumbado tras el ejercicio?*” (“To what degree does your dog show stiffness in the affected leg after a “lie down” following exercise?”), should be changed from “*Rigidez severa*” (“Severe stiffness”) and “*Rigidez extrema*” (“Extreme stiffness”) to “*Rigidez grave*” (“Serious stiffness”) and “*Rigidez muy grave*” (“Very serious stiffness”), respectively. In this case, in the end it was decided to modify only the option “*Rigidez severa*” to “*Rigidez grave*,” since although they are very similar terms and it is not a problem of understanding, grammatically it is more correct to use the adjective “*grave*” in this context than “*severa*” (harsh in treatment or punishment, or rigid in the observance of a rule). Another participant suggested modifying the answer options “*No muy activo*” in question 6 “At exercise, how active is your dog” and “*No muy interesado*” in question 7 “How interested is your dog in exercising” to “*Poco activo*” and “*Poco interesado*,” respectively. The researchers accepted this modification because, although there were no problems of understanding, both options are correct and mean the same thing, but in Spanish this grammatical construction (“*Poco interesado*” and “*Poco activo*”) is more common than the use of “*No muy activo*” and “*No muy interesado*,” which would make them easier to read.

In total, 12 questions and answers were modified after the cognitive analysis. Annex III shows all comments and suggestions from the cognitive analysis, as well as the modifications made.

Once a revised version was produced based on the previous results, a second cognitive analysis including 17 participants was performed. Of these individuals, 10 were women and 7 were men. The age ranges included were: ≤ 29 years (*n* = 2), 30–39 years (*n* = 5), 40–49 years (*n* = 3), 50–59 years (*n* = 4), ≥60 years (*n* = 3). The education level considered included primary school (*n* = 0), secondary school (*n* = 1), high school (*n* = 2), and postgraduate studies (*n* = 14). In general, all of them understood all the questions and answer options, although there were two participants who suggested some changes in terms of modifying a question to make it more understandable. These changes were discarded as they did not pose a problem of understanding. One of them commented that he could not see any relationship between question 3 of the lifestyle section: “¿*Qué tipo de actividad es esta*?” (What type of exercise is this?) with the answer options: “*Siempre con correa*,” “*Casi siempre con correa*,” “*Casi siempre sin correa*,” “*Siempre sin correa*,” and “*Trabajando (perro de trabajo*)” and that she would rephrase the question as “¿*Cómo pasea usted a su perro*? (How you walk your dog?).” This question already raised understanding issues in the first cognitive analysis and had been modified previously. Considering all the comments and despite the fact that the back-translation matched the original version, the research team decided to rewrite the question to “¿*Cómo hace esta actividad*?” Although it differed from the English version (the English translation would be: “How does he/her do this activity?”), this small modification had to be made because if it were translated literally, it would be confusing in Spanish. The Spanish translation of LOAD can be downloaded at https://assets.elanco.com/0cec44ed-3eaa-0009-2029-666567e7e4de/2f12e790-db29-46cc-bed6-4c914f776af9/Spanish_LOAD_24.pdf.

## 4 Discussion

A translation and linguistic validation of the LOAD into Spanish has been produced. The LOAD is an OROM originally written in English, with only a validated translation into Portuguese (4). The translation has been cognitively tested by owners and the necessary adjustments to the test were made. To implement this tool globally and make it accessible to all veterinarians, translation into different languages, including Spanish, is crucial. This allows its use by a much broader community of dog owners while maintaining the conceptual integrity of the original version.

The observed discrepancies between translations and back-translations mainly focused on choosing a more suitable term that captured the concept or nuance of the original. For example, the term “fair” has various meanings, and within the context of other responses, “*aceptable*” (acceptable) fits better. Another example is the translation of the term “poor”' which lacks the moral connotation of “bad” when translated into Spanish, this moral connotation does not always apply, and the use of “*malo*” better reproduces the original concept, preventing a confusing translation through a mere transliteration. Overall discrepancies were relatively few and easy to review; suggesting the original survey in English was straightforward and easily understandable. More discrepancies were observed in the back translation. However, the English native linguist was not a veterinarian and thus unlikely to use exactly the same technical terms or sentences, although most discrepancies were irrelevant.

A high number of responses were gathered from owners from different gender, ages, and cultural background. Reported doubts or suggestions in the cognitive evaluation were minimal, which was expected as the survey posed short and relatively simple questions, along with straightforward response options. One translation drew attention from several respondents, likely due to being an uncommon term in Spanish. Specifically, the term “working” (“*trabajando*”) does not usually apply to dogs engaged in professional activities, and lead to comprehension doubts among respondents in the cognitive evaluation. In this case, and not having identified an easily, unique, understandable equivalent term, it was considered to add a brief explanation (“working dogs” or “*perros de trabajo*”). This simple explanation facilitated understandability from these same respondents afterwards. Perhaps an example of these activities would have been more intuitive, but in this case, a more faithful translation to the original was preferred, avoiding expanding the explanation. Another question that proved to be confusing was number 3 in the lifestyle section, “¿*Cómo hace esta actividad?*” (“What type of exercise is this?”). It had to be translated again several times as several participants did not understand the connexion with other previous questions and did not see any connexion between the question and the answer options. The research team has opted to rephrase the content from the original version in a manner that ensures it is easily comprehensible to the owners. It was not considered that this would affect the results or significantly modify the questionnaire, since, although the question does not match exactly and is not a literal translation, the meaning and sense of the question is the same as in the English version. It should be noted that the current translation has been performed to meet Spain's cultural requirements. Therefore, minor adjustments may be considered to cope with different Spanish-speaking countries like those in South and Central America, but also in North America. This may include not only the use of country-specific words but also additional adjustments such as the use of miles instead of kilometres.

To achieve a linguistically validated version of the LOAD, recommendations and guidelines published (12, 14–16) were followed. Two native Spanish-speaking linguists were selected for direct translations, one with technical knowledge and familiarity with health sciences, while the other had no training on health sciences. Although guidelines recommend that direct translations should be carried out by healthcare professionals, a second non-technical linguist was included by the research team, considering better reflected the average pet owner in clinical practise. While this decision may have interfered with the translation process, it was considered that the unified or reconciliation translation by veterinarians, has not hindered or prevented a reliable translation of the LOAD into Spanish. In the Portuguese translation of the LOAD (4), a team of veterinarians performed an independent translation without considering a linguist with no medical background. Another limitation of this study is that all participants were from a specific geographic area (Madrid province), and participants from other areas of Spain were not included, potentially introducing sociocultural variables into the results.

Translation guidelines do not specify the characteristics of linguists needed for translations (they should be native speakers of the target language). The linguists in this study did not have specific qualifications or training, which could have influenced the results by overlooking inconsistencies or grammatical errors in the translation. This could have been avoided by including a Spanish or English philologist. On the other hand, the use of a relatively technical yet simple language of OA in dogs suggests that this factor might have less relevance.

## 5 Conclusion

In conclusion, this study provides a linguistically validated version of the LOAD in Spanish, promoting its use by Spanish-speaking veterinarians and researchers for the assessment and management of chronic pain in dogs. The next step will be to conduct psychometric validation of the Spanish translation of the LOAD to ensure greater reliability and validity.

## Data availability statement

The original contributions presented in the study are included in the article/Supplementary material, further inquiries can be directed to the corresponding author.

## Ethics statement

The studies involving humans were approved by Research Ethics and Biosafety Committees Universidad Complutense de Madrid (Ref: CE_20230511-02_SAL, 11 May 2023). The studies were conducted in accordance with the local legislation and institutional requirements. The participants provided their written informed consent to participate in this study. Written informed consent was obtained from the individual(s) for the publication of any potentially identifiable images or data included in this article.

## Author contributions

MO: Writing – original draft, Writing – review & editing. MC: Writing – review & editing, Conceptualisation. IG: Writing – review & editing, Conceptualisation.
