# Supplementary material for: Translation and linguistic validation into Spanish of the Owner-Reported Outcome Measure “Liverpool Osteoarthritis in Dogs”
Source: Front Vet Sci. 2024 Feb 20;11:1360926. doi: 10.3389/fvets.2024.1360926 (PMC10912287; doi:10.3389/fvets.2024.1360926)
Supplement: Supplementary file 1 [file Table_1.DOCX]

Supplementary Material

**ANNEX I. Discrepancies between independent translations and unified translation.**

| **Original** | **Independent translation 1** | **Independent translation 2** | **Unified translation** |
| --- | --- | --- | --- |
| *Most of the questions are fairly simple. It is important that you only check one box per question except where otherwise requested (e.g. Question 4 under Lifestyle)* | La mayoría de las preguntas son bastante simples. Es importante que solo marque una casilla por pregunta excepto donde se solicite lo contrario (por ejemplo, en la pregunta 4 en estilo de vida) | La mayoría de las preguntas son simples. Es importante que marque únicamente una casilla por pregunta excepto donde se solicite lo contrario (por ejemplo, en la pregunta 4 en estilo de vida) | La mayoría de las preguntas son bastante sencillas. Es importante que marque solo una casilla por pregunta, salvo que se le pida lo contrario (por ejemplo, la pregunta 4 sobre estilo de vida) |
| *1. How long has your pet been suffering with his/her mobility problem?* | 1. ¿Cuánto tiempo lleva su mascota con problemas de movilidad? | 1. ¿Cuánto tiempo lleva su mascota con su problema de movilidad? | 1. ¿Cuánto tiempo lleva su mascota padeciendo su problema de movilidad? |
| *3. If you can, please list any medications that your pet is currently receiving, stating when she/he received the last dose of each* | 3. Si puede por favor enumere cualquier medicamento que esté tomando su mascota y cuándo fue la última dosis de cada uno | 3. Enumere si puede por favor, cualquier medicamento que esté tomando su mascota y cuándo fue la última dosis de cada uno | 3. Si puede, por favor haga una lista de los medicamentos que esté tomando su mascota, indicando cuándo recibió la última dosis de cada uno |
| *0-0.6 miles/0.6-1.2 miles/1.2-1.9 miles/1.9-2.5 miles/More than 2.5 miles* | 0-1 km/1-2 km/2-3 km/3-4 km/Más de 4 km | 0-0,6 millas/0,6-1.2 millas/1,2-1,9 millas/1,9-2,5 millas/Más de 2,5 millas | 0-1 km/1-2 km/2-3 km/3-4 km/Más de 4 km |
| *4. Are there particular days of the week upon which your dog has significantly more exercise? (Check more than one box if necessary)* | 4. ¿Hay días concretos de la semana en los que su perro hace mucho más ejercicio? (marcar más de una casilla si es necesario) | 4. ¿Hay días concretos de la semana en los que su perro hace más ejercicio significativamente? (marcar más de una casilla si es necesario) | 4. ¿Hay días concretos de la semana en los que su perro hace bastante más ejercicio? (Marque más de una casilla si es necesario) |
| *On level grass/Over rough ground* | Sobre hierba nivelada/Sobre terreno accidentado | Sobre hierba plana/Sobre terreno irregular | Sobre césped llano/Sobre terreno accidentado |
| *1. How is your dog’s mobility in general?* | 1. ¿Cómo es en general la movilidad de tu perro? | 1. ¿Cómo es en general la movilidad de su perro? | 1. ¿Cómo es la movilidad de su perro en general? |
| *2. How disabled is your dog by his/her lameness* | 2. ¿Cuál es el grado de discapacidad de tu perro por su cojera? | 2. ¿Cuál es el grado de discapacidad de su perro por su cojera? | 2. ¿Cuál es el grado de discapacidad que produce la cojera de su perro? |
| *Extremely disabled* | Extremadamente incapacitante | Discapacidad extrema | Discapacidad extrema |
| *3. How active is your dog?* | 3. ¿Cómo de activo es tu perro? | 3. ¿Cómo de activo es su perro? | 3. ¿Cómo de activo es su perro? |
| *4. What is the effect of cold, damp weather on your dog’s lameness* | 4. ¿Qué efecto tiene el frío y el clima húmedo en la cojera de tu perro? | 4. ¿Qué efecto tiene el frío y el clima húmedo en la cojera de su perro? | 4. ¿Qué efecto tiene el frío y el clima húmedo en la cojera de su perro? |
| *5. To what degree does your dog show stiffness in the affected leg after a “lie down”?* | 5. ¿Hasta qué punto tu perro muestra rigidez en la pata afectada después de estar tumbado? | 5. ¿Hasta qué punto su perro muestra rigidez en la pata afectada después de tumbarse? | 5. ¿Hasta qué punto su perro muestra rigidez en la extremidad afectada después de estar tumbado? |
| *Mild stiffness/Moderate stiffness/Severe stiffness/Extreme stiffness* | Rigidez leve/Rigidez moderada/Rigidez severa/Rigidez extrema | Leve rigidez/Moderada rigidez/Severa Rigidez/Extrema rigidez | Rigidez leve/Rigidez moderada/Rigidez severa/Rigidez extrema |
| *6. At exercise, how active is your dog?* | 6. En el ejercicio, ¿cómo es la actividad de tu perro? | 6. En el ejercicio, ¿cómo es la actividad de su perro? | 6. Cuando hace ejercicio, ¿cómo de activo es su perro? |
| *8. How would you rate your dog’s ability to exercise?* | 8. ¿Cómo calificaría la capacidad de tu perro para hacer ejercicio? | 8. ¿Cómo calificaría la capacidad de su perro para hacer ejercicio? | 8. ¿Cómo calificaría la capacidad de su perro para hacer ejercicio? |
| *9. What overall effect does exercise have on your dog’s lameness?* | 9. ¿Qué efecto global tiene el ejercicio sobre la cojera de tu perro? | 9. ¿Qué efecto global tiene el ejercicio sobre la cojera de su perro? | 9. ¿Qué efecto global tiene el ejercicio sobre la cojera de su perro? |
| *10. How often does your dog rest (stop/sit down) during exercise?* | 10. ¿Con qué frecuencia tu perro descansa durante el ejercicio (se para o se sienta)? | 10. ¿Con qué frecuencia su perro descansa durante el ejercicio (se para o se sienta)? | 10. ¿Con qué frecuencia descansa su perro (se para/se sienta) durante el ejercicio? |
| *11. What is the effect of cold, damp weather on your pet’s ability to exercise?* | 11. ¿Qué efecto tiene el frío y el clima húmedo sobre la capacidad de tu mascota para hacer ejercicio? | 11. ¿Qué efecto tiene el frio y el clima húmedo sobre la capacidad de su mascota para hacer ejercicio? | 11. ¿Qué efecto tiene el clima frío y húmedo en la capacidad de su mascota para hacer ejercicio? |
| *12. To what degree does your dog show stiffness in the affected leg after a “lie down”?* | 12. ¿Hasta qué punto tu perro muestra rigidez en la extremidad afectada después de haber estado tumbado después del ejercicio? | 12. ¿Hasta qué punto tu perro muestra rigidez en la extremidad afectada después de haber estado tumbado tras el ejercicio? | 12. ¿Hasta qué punto su perro muestra rigidez en la extremidad afectada después de haber estado tumbado tras el ejercicio? |
| *Moderate stiffness/Severe stiffness* | Rigidez moderada/Rigidez severa | Moderada rigidez/Severa rigidez | Rigidez moderada/Rigidez severa |
| *13. What is the effect on your dog’s lameness on his/her ability to exercise?* | 13. ¿Cuál es el efecto de la cojera de tu perro en su capacidad para hacer ejercicio? | 13. ¿Cuál es el efecto de la cojera de su perro en su capacidad para hacer ejercicio? | 13. ¿Qué efecto tiene la cojera de su perro en su capacidad para hacer ejercicio? |

**ANNEX II. Discrepancies between the original version and the back-translation and the modifications made.**

| **Original** | **Unified translation** | **Back-translation** | **Review by the research team and a native English-speaking linguist** | **Modifications made** |
| --- | --- | --- | --- | --- |
| *For office use only* | Sólo para uso clínico | *Only for clinic use* | The researchers decided to replace the term "clínico" with "administrativo" to make the translation more faithful to the original version and to indicate that the questionnaire is only to be used by the staff providing the questionnaire to the patient | Sólo para uso administrativo |
| *Owner questionnaire for dogs with mobility problems* | Cuestionario para propietarios de perros con problemas de movilidad | *Questionnaire for owners of dogs with movement difficulties/problems* | Although the back-translation is not exactly the same as the original version, no modification was considered, as there is no difference in the meaning of the sentence | None |
| *Thank you for agreeing to complete this questionnaire* | Gracias por acceder a completar este cuestionario | *Thank you for completing this questionnaire* |  |  |
| *Your assistance in this endeavour will enable us to gather valuable information about your pet, and is a vital component in our ongoing quest to combat painful and debilitating diseases such as arthritis. It is important that all questions are answered to the best of your ability and if you have a question regarding the questionnaire, please contact a health care member from your veterinary clinic* | Su ayuda en esta tarea nos permitirá recopilar información valiosa sobre su mascota, y es un componente vital en nuestra búsqueda continua para luchar contra enfermedades dolorosas y debilitantes como la artritis. Es importante que responda a todas las preguntas lo mejor que pueda y, si tiene alguna duda sobre el cuestionario, póngase en contacto con un miembro del personal sanitario de su clínica veterinaria | *Your help in this task will allow us to gather valuable information about your pet and is a vital component in our continuous search in the fight against painful and weakening diseases like arthritis. It is important for you to respond to all the questions the best you can, and, if you have any doubts about the questionnaire/questions, please contact a member of the sanitary staff of your veterinary clinic* |  |  |
| *Most of the questions are fairly simple. It is important that you only check one box per question except where otherwise requested (e.g. Question 4 under Lifestyle)* | La mayoría de las preguntas son bastante sencillas. Es importante que marque sólo una casilla por pregunta, salvo que se le pida lo contrario (por ejemplo, la pregunta 4 sobre estilo de vida) | *Most of the questions are fairly simple. It is important for you to check only one box per question, unless you are asked to do the contrary (for example, question 4 on Life Style)* |  |  |
| *Today’s date* | Fecha actual | *Present date* |  |  |
| *Reference limb* | Extremidad afectada | *Affected limb* | The research team decided to modify the term "afectada" to "considerada", as the former assumes that there is a problem with the limb and that it suffers from some problem or pathology, while "considerada" indicates that it is the limb that is being referred to and assessed | Extremidad considerada |
| *Background* | Antecedentes | *Antecedents* | Although the back-translation is not exactly the same as the original version, no modification was made by considering "background" and "antecedents" as synonyms and referring to the patient's clinical history | None |
| *1. How long has your pet been suffering with his/her mobility problem?* | 1. ¿Cuánto tiempo lleva su mascota padeciendo su problema de movilidad? | *1. How long has your pet had/difficulty moving?/ his/her mobility problem?* | Although the back-translation is not exactly the same as the original version, no modification was considered, as there was no difference in the meaning of the question |  |
| *Up to 6 months* | Menos de 6 meses | *Less than 6 months* | The research team felt that there was some difference in meaning, as "up to 6 months" indicates a period of time up to and including 6 months, while "less than 6 months" indicates a period of time that does not include 6 months. The translation was changed to "Hasta 6 meses" | Hasta 6 meses |
| *2. Has your dog been diagnosed as suffering from any other problems in addition to his/her orthopedic disease* | 2. ¿Se le ha diagnosticado a su perro algún otro problema además de su enfermedad ortopédica? | *2. Has your pet been diagnosed with any other problem beyond its orthopedic disease?* | Despite finding no significant differences in the meaning of the phrase, the researchers decided to make a slight modification and include the term "padece", which translates as "suffering", to make it more similar to the original version | 2. ¿Se le ha diagnosticado a su perro, o padece, algún otro problema además de su enfermedad ortopédica? |
| *Please list these if you can* | Enumérelos si puede | *List them if you can* | Although the back-translation is not exactly the same as the original version, no modification was considered, as there was no difference in the meaning of the question | None |
| *3. If you can, please list any medications that your pet is currently receiving, stating when he/she received the last dose of each* | 3. Si puede, por favor haga una lista de los medicamentos que esté tomando su mascota, indicando cuándo recibió la última dosis de cada uno | *3. If you can, make a list of the medications your dog is taking, indicating when they received their last dose of each one?* |  |  |
| *1. In the last week, on average, how far has your dog exercised each day?* | 1. En la última semana, de promedio, ¿cuánto ejercicio ha hecho su perro cada día? | *1. In the last week, on average, how much exercise has your dog done each day?* | The researchers decided to rewrite the question, as they are asking about the distance the dog has travelled and not the amount of exercise it has done | 1. En la última semana, de promedio ¿cuánta distancia ha recorrido su perro cada día? |
| *More than 2.5 miles* | Más de 4 km | *Over 2.5 miles* | Although the back-translation is not exactly the same as the original version, no modification was made as "more" and "over" are considered to be synonyms | None |
| *3. What type of exercise is this?* | 3. ¿Qué tipo de ejercicio es este? | *3. What kind of exercise is it/How do you walk it?* | Although the back-translation matched the original version, it was too literal a translation and could pose a comprehension problem by not correctly expressing the purpose of the question. The researchers decided to rewrite the question to "¿Cómo hace el ejercicio?", even though the back-translation did not exactly match the original, it was thought to reflect the question more comprehensibly | 3. ¿Cómo hace el ejercicio? |
| *Always on leash* | Siempre con correa | *Always on a lead* | Although the back-translation is not exactly the same as the original version, no modification was made as "leash" and "lead" are considered to be synonyms | None |
| *Mostly on leash* | Casi siempre con correa | *Nearly always on a lead* | No change was made as "leash/lead" and "mostly/nearly always" were considered to be synonyms |  |
| *Mostly off leash* | Casi siempre sin correa | *Nearly always without a lead* |  |  |
| *Always off leash* | Siempre sin correa | *Always without a lead* |  |  |
| *4. Are there particular days of the week upon which your dog has significantly more exercise? (Check more than one box if necessary)* | 4. ¿Hay días concretos de la semana en los que su perro hace bastante más ejercicio? (Marque más de una casilla si es necesario) | *4. Are there days of the week on which your dog has significantly more exercise than others? (Mark more than one box if necessary)* | Although the back-translation is not exactly the same as the original version, no modification was considered, as there was no difference in the meaning of the question |  |
| *5. On what sort of terrain does your dog most often exercise?* | 5. ¿Sobre qué tipo de terreno su perro hace ejercicio con más frecuencia? | *5. On what kind of terrain does your dog exercise most frequently? /What kind of ground do you walk your dog on?* |  |  |
| *On level grass* | Sobre césped llano | *Smooth/flat lawn* | Although the back-translation is not exactly the same as the original version, no modification was considered, as there was no difference in the meaning of the answer |  |
| *In woodland* | En el bosque | *In the Woods* | Although the back-translation was very similar to the original version and did not change the meaning of the answer, it was finally decided to change the translation to "Sobre terreno boscoso” since it is referring to a type of terrain and by appearing as "En el bosque" it would be referring to a specific location | Sobre terreno boscoso |
| *Over rough ground* | Sobre terreno accidentado | *Broken ground* | The researchers considered translating it back and replacing the term "accidentado" with "irregular" | Sobre terreno irregular |
| *6. At exercise, how is your dog handled?* | 6. Durante el ejercicio, ¿cómo lleva a su perro? | *6. During exercise, how do you lead your dog?* | Although the back-translation is not exactly the same as the original version, no modification was considered, as there was no difference in the meaning of the question | None |
| *Walk on leash* | Camina con correa | *He walks on a lead* | No change was made as "leash" and "lead" were considered to be synonymous |  |
| *Walk off leash* | Camina sin correa | *He walks off a lead* |  |  |
| *7. Who limits the extent to which your dog exercises?* | 7. ¿Quién limita cuánto ejercicio hace su perro? | *7. Who limits how much exercise your dog gets?* | Although the back-translation is not exactly the same as the original version, no modification was considered, as there was no difference in the meaning of the question |  |
| *Generally* | En general | *In general* | Although "generally" and "in general" are synonyms, the researchers decided to translate it back to "generalmente" as there is a slight difference and "generalmente" is more commonly used to describe life habits and trends than "en general" | Generalmente |
| *Fair* | Aceptable | *Acceptable* | No change was made as "fair" and "acceptable" were considered to be synonymous in this context | None |
| *2. How disabled is your dog by his/her lameness* | 2. ¿Cuál es el grado de discapacidad que produce la cojera de su perro? | *2. What is the degree of discapacity produced by your dog’s limp?* | The research team decided to rewrite the question, as the answer options ("Not at all disabled", "Slightly disabled", etc.) do not match grammatically with the question (in this case the answers should be rewritten as: "No degree of disability", "Mild degree of disability", etc.) and would be a relevant change from the original | 2. ¿Cómo de discapacitado está su perro por la cojera? |
| *Not at all disabled* | Ninguna discapacidad | *No discapacity* | No modification was made as "disabled" and "discapacity" are considered synonyms | None |
| *Slightly disabled* | Discapacidad leve | *Slight discapacity* | It was decided to make a minor modification and replace the adjective "leve" with the adverb "levemente", to match the English version. No further modification was made as "disabled" and "discapacity" were considered to be synonyms | Levemente discapacitado |
| *Moderately disabled* | Discapacidad moderada | *Moderate discapacity* | It was decided to make a minor modification and replace the adjective "moderada" with the adverb "moderadamente", to match the English version. No further modification was made as "disabled" and "discapacity" were considered to be synonyms | Moderadamente discapacitado |
| *Severely disabled* | Discapacidad severa | *Severe discapacity* | It was decided to make a modification and replace the adjective "severa" with the adverb "severamente", to match the English version. No further modification was made as "disabled" and "discapacity" were considered to be synonyms. After cognitive debriefing it was finally decided to change the term “severamente” to “gravemente” (see below in the cognitive debriefing) | Gravemente discapacitado |
| *Extremely disabled* | Discapacidad extrema | *Extreme discapacity* | It was decided to make a minor modification and replace the adjective "extrema" with the adverb "extremadamente", to match the English version. No further modification was made as "disabled" and "discapacity" were considered to be synonyms | Extremadamente discapacitado |
| *4. What is the effect of cold, damp weather on your dog’s lameness?* | 4. ¿Qué efecto tiene el clima frío y húmedo en la cojera de su perro? | *4. What effect do the cold and damp weather have on your dog’s limp?* | Although the back-translation is not exactly the same as the original version, no modification was made, as there is no difference in the meaning of the question and "lameness" and "limp" are considered to be synonyms | None |
| *Mild effect* | Efecto leve | *Slight* | Although the back-translation did not exactly match the original version, no modifications were made. The native linguist involved in this work considered translating the terms "leve" and "moderado" as "slight" and "mild", respectively, and the clinical terms "mild-moderate-severe" are usually translated as "leve-moderado-grave" in the veterinary clinical setting and it was decided to keep this criterion |  |
| *Moderate effect* | Efecto moderado | *Mild* |  |  |
| *5. To what degree does your dog show stiffness in the affected leg after a “lie down”?* | 5. ¿Hasta qué punto su perro muestra rigidez en la extremidad afectada después de estar tumbado? | *5. To what extent does your dog display rigidity in the affected limb after being lying down?* | Only the term "punto" was changed to "grado" to match the original version. The rest of the question remained the same as "stiffness/rigidity", "show/display" and "leg/limb" were considered to be synonyms | 5. ¿Hasta qué grado su perro muestra rigidez en la extremidad afectada después de estar tumbado? |
| *No stiffness* | Sin rigidez | *No rigidity* | No modification was made, as "stiffness" and "rigidity" were considered to be synonymous | None |
| *Mild stiffness* | Rigidez leve | *Slight rigidity* | Although the back-translation did not exactly match the original version, no modifications were made. The native linguist involved in this work considered translating the terms "leve" and "moderado" as "slight" and "mild", respectively, and the clinical terms "mild-moderate-severe" are normally translated as "leve-moderado-grave" in the veterinary clinical setting, and this criterion has been retained |  |
| *Moderate stiffness* | Rigidez moderada | *Mild rigidity* |  |  |
| *Extreme stiffness* | Rigidez extrema | *Extreme rigidity* | No modification was made, as "stiffness" and "rigidity" were considered to be synonymous |  |
| *At exercise* | En ejercicio | *When exercising* | Although there was no difference in the meaning of the statement, the researchers decided to translate it again, to make it sound more natural and to make it easier to understand in Spanish | Durante el ejercicio |
| *Fairly active* | Aceptablemente activo | *Acceptably/moderately active* | No amendment was made, as "fairly" and "acceptably/moderately" are considered to be synonymous | None |
| *Fairly interested* | Bastante interesado | *Acceptably interested* | Although "fairly" and "acceptably" are considered synonymous, the research team decided to change the term "bastante" to "aceptablemente". The reason for this is that in the previous question the term "fairly" was translated as "aceptablemente" and here it was translated as "bastante" and, although they mean the same thing and can be used interchangeably, it was decided to keep the same translation in both cases in order to avoid using different terms for the same meaning that could lead to greater confusion or difficulty in understanding the questions and answers | Aceptablemente interesado |
| *8. How would you rate your dog’s ability to exercise?* | 8. ¿Cómo calificaría la capacidad de su perro para hacer ejercicio? | *8. How would you grade your dog’s ability to exercise?* | No change was made, as "rate" and "grade" were considered to be synonymous | None |
| *Fair* | Aceptable | *Acceptable* | No change was made as "fair" and "acceptable" were considered to be synonymous |  |
| *9. What overall effect does exercise have on your dog’s lameness?* | 9. ¿Qué efecto global tiene el ejercicio sobre la cojera de su perro? | *9. What global effect does exercise have on your dog’s limp?* | No change was made as "overall/global" and "lameness/limp" were considered to be synonymous |  |
| *Hardly ever* | Casi nunca | *Almost never* | No change was made as "hardly ever" and "almost never" were considered to be synonyms |  |
| *11. What is the effect of cold, damp weather on your pet’s ability to exercise?* | 10. ¿Qué efecto tiene el clima frío y húmedo en la capacidad de su mascota para hacer ejercicio? | *10. What effect does a cold damp climate have on your dog’s capacity for exercise?* | Although the back-translation is not exactly the same as the original version, no modification was made, as there is no difference in the meaning of the question |  |
| *12. To what degree does your dog show stiffness in the affected leg after a “lie down” following exercise?* | 12. ¿Hasta qué punto su perro muestra rigidez en la extremidad afectada después de haber estado tumbado tras el ejercicio? | *12. To what extent does your dog exhibit rigidity in the affected leg/limn after lying down after exercise?* | Only the term "punto" was changed to "grado" to match the original version. The rest of the question remained the same as, despite some differences, the meaning is the same | 12. ¿Hasta qué grado su perro muestra rigidez en la extremidad afectada después de haber estado tumbado tras el ejercicio? |
| *13. What is the effect of your dog’s lameness on his/her ability to exercise?* | 13. ¿Qué efecto tiene la cojera de su perro en su capacidad para hacer ejercicio? | *13. What effect does your dog’s limp have on his capacity for exercise?* | Although the back-translation is not exactly the same as the original version, no modification was made, as there is no difference in the meaning of the statement | None |
| *Thank you once again for completing this questionnaire* | Gracias una vez más por completar este cuestionario | *Once again, thank you for filling in this questionnaire* |  |  |
| *Please return the form to a staff member* | Por favor, devuelva el formulario a un miembro del personal | *Please return the questionnaire to a staff member* |  |  |

**ANNEX III. Cognitive debriefing and final review**

| **Original** | **Unified translation** | **Cognitive debriefing** | **Modifications made** | **Second cognitive debriefing** |
| --- | --- | --- | --- | --- |
| *1. In the last week, on average, how far has your dog exercised each day?* | 1. En la última semana, de promedio, ¿cuánta distancia ha recorrido su perro cada día? | One participant considered the question weird and would change the term "¿cuánta distancia...?" to "¿qué distancia...?". The researchers considered that this was a small modification that did not alter the meaning of the question, and that, although both options are grammatically correct, the second option was more natural and facilitated reading comprehension, so they decided to include this change in the final version in Spanish | 1. En la última semana, de promedio, ¿qué distancia ha recorrido su perro cada día? | None of the participants had any comprehension issues |
| *3. What type of exercise is this?* | 3. ¿Cómo hace el ejercicio? | There were three participants who had problems with this question, and it was not clear what it referred to. One indicated that they did not understand if it was referring to the previous questions (1. “En la última semana, de promedio, ¿qué distancia ha recorrido su perro cada día?” y 2. “En la última semana, de promedio ¿cuántos paseos ha dado su perro al día?”) o a algún otro tipo de actividad específica. or to some other specific activity. Another commented that it closely resembled question 6. "Durante el ejercicio, ¿cómo lleva a su perro?" and did not understand the differences between the two questions, suggesting joining both. The research team analysed this question and decided to translate it again to make it more clear and referring to the previous questions. The term "ejercicio" was changed to "actividad" (although in Spanish there are certain differences, in English both terms, translated as “exercise” and “activity”, are considered synonymous) | 3. ¿Qué tipo de actividad es esta? | One of the respondents mentioned that they did not understand the question well and saw no consistency with the response options. This respondent suggested rephrasing the question to "¿Cómo pasea usted a su perro?”. Finally, the researchers decided to rephrase the question once again to "¿Cómo hace esta actividad?". Although this differs from the original version (in English, it would be "How does he/she do/perform this activity?"), a more literal translation into Spanish would pose comprehension difficulties. Despite this, the meaning of the question remained the same as in English. |
| *Working* | Trabajando | There were 9 participants who had difficulties with question number 3: "¿Cómo hace el ejercicio?", in relation to the answer option "Trabajando", indicating that they did not understand this concept". The research team decided to add in the final translation a comment: "Trabajando (perro de trabajo)" to explain that the physical activity he performs is done as a working dog | Trabajando (perro de trabajo) | None of the participants had any comprehension issues |
| *6. At exercise, how is your dog handled?* | 6. Durante el ejercicio, ¿cómo lleva a su perro? | One of the respondents proposed to change this question to "Durante el ejercicio, ¿cómo va su perro?", stating that there was a grammatical inconsistency between the subject of the question (the owner) and the subject of the answers (the dog), i.e. the question asks how the owner is leading his dog (on leash, off leash, etc.), but the answers refer to how the dog is doing (walking, trotting, etc.). The research team decided to modify this question as suggested by the participant.  Another participant suggested adding a multiple response option to this question. This was not considered as it would mean modifying the questionnaire, which could have repercussions for the interpretation of the results | 6. Cuando hace ejercicio, ¿cómo va su perro? |  |
| *Poor* | Pobre | There were two participants who suggested that in questions 1. "¿Cómo es la movilidad de su perro en general?" and 6. "Cuando hace ejercicio, ¿cómo de activo es su perro?" despite correctly understanding the response options, they would change the options "Pobre" and "Muy pobre" to "Mala" and "Muy mala" respectively. Another participant indicated that the terms "Pobre" and "Muy pobre" were not very clear. The research team decided to change the terms "Pobre" and "Muy pobre" to "Mala" and "Muy mala" in both questions 1 and 6, since in Spanish the term "Pobre" are usually used as an adjective to indicate that something is humble or scarce in economic terms, while "Mala" are adjectives used to indicate a negative value | Mala |  |
| *Very Poor* | Muy pobre |  | Muy mala |  |
| *Severely disabled* | Severamente discapacitado | Although there were no comprehension problems and the back-translation was practically the same, it was decided to replace the term "severamente" with "gravemente", since although both terms are correct, in Spanish it is more common within non knowledgeable persons to use the adjective “grave” in this context. | Gravemente discapacitado | None of the participants had any comprehension issues |
| *Severe stiffness* | Rigidez severa | One of the participants suggested changing the terms "severa" and "extrema" to "grave" and "muy grave". Although there were no comprehension problems and the back-translation was practically the same, it was decided to replace the term "severo" with "grave", since although both terms are correct, in Spanish it is more common within non knowledgeable persons to use the adjective “grave” in this context. However, it was decided to keep the term "extrema" for "muy grave" as it did not pose any comprehension problems. | Rigidez grave |  |
| *Extreme stiffness* | Rigidez extrema |  | None |  |
| *2. Has your dog been diagnosed as suffering from any other problems in addition to his/her orthopedic disease?* | 2. ¿Se le ha diagnosticado a su perro, o padece, algún otro problema además de su enfermedad ortopédica? | One participant suggested changing "enfermedad ortopédica" to "enfermedad osteoarticular o dolorosa" and another indicated that, although the term was understood, he would add an example of orthopaedic disease to make it clearer. As this was not a problem of comprehension and in order to maintain a more faithful translation of the original document, thus avoiding unnecessary additions, no changes were made |  |  |
| *3. How active is your dog?* | 3. ¿Cómo de activo es su perro? | One participant indicated that he found the term "activo" ambiguous in these two questions and that it would need to be defined. As none of the other participants had problems understanding the questions, it was not considered necessary to specify anything further, because it is also understood that in both questions the owner is being asked to define what he/she considers to be active (extremely active, very active, etc.) |  |  |
| *6. At exercise, how active is your dog?* | 6. Cuando hace ejercicio, ¿cómo de activo es su perro? |  |  |  |
| *5. To what degree does your dog show stiffness in the affected leg after a “lie down”?* | 5. ¿Hasta qué grado su perro muestra rigidez en la extremidad afectada después de estar tumbado? | One respondent proposed changing the question to "¿Hasta qué grado su perro muestra rigidez en la extremidad afectada al levantarse después de estar tumbado?". The researchers rejected this change, as it would change the meaning of the question, which could alter the results and the final score, as the wording would be assessing the dog's difficulty only in getting up, and not how the dog acts after lying down, regardless of whether it had difficulty getting up or not | None | Although there were no comprehension problems, one of the participants in the second cognitive analysis suggested rephrasing the question to "¿Hasta qué grado muestra rigidez su perro en la extremidad afectada después de estar tumbado?”. This change was not considered as there were no comprehension problems and no further comments |
| *On street* | En la calle | One respondent proposed adding "En la calle o asfalto", but the change was not considered as it did not pose a problem of comprehension | None |  |
| *Over rough ground* | Sobre terreno irregular | One participant indicated that he found this answer confusing, as the other options (“En el bosque”, “En la calle” ...) can also be irregular. The research team decided to re-translate it as "Sobre terreno accidentado", to better differentiate the response options and to indicate that this is a barren and complicated terrain. | Sobre terreno accidentado | None of the participants had any comprehension issues |
| *10. How often does your dog rest (stop/sit down) during exercise?*  *Never/Hardly ever/Occasionally/Frequently/Very frequently* | 10. ¿Con qué frecuencia descansa su perro (se para/se sienta) durante el ejercicio?  Nunca/Casi nunca/Ocasionalmente/Frecuentemente/Muy frecuentemente | One of the participants suggested adding a score to the response options: "Ocasionalmente (2-3 veces), Frecuentemente (4 veces), etc." The researchers rejected this because it would mean modifying the original version | None |  |
| *4. Are there particular days of the week upon which your dog has significantly more exercise? (Check more than one box if necessary)*   - *Monday* - *Tuesday* - *Wednesday* - *Thursday* - *Friday* - *Saturday* - *Sunday* | 4. ¿Hay días concretos de la semana en los que su perro hace bastante más ejercicio? (Marque más de una casilla si es necesario)   - Lunes - Martes - Miércoles - Jueves - Viernes - Sábado - Domingo | One of the participants commented that, in computer science, round boxes are used in case of single response options, while the square is used for multiple response options. No changes were made to maintain the same format as the original version |  |  |
| *Not very active* | No muy activo | One respondent suggested changing the response options "No muy activo" and "No muy interesado" to "Poco activo" and "Poco interesado". The researchers accepted this change, as although it was not a problem of comprehension and both terms are correct, in Spanish it is more common and easier to read "Poco" than "No muy..." | Poco activo | None of the participants had any comprehension issues |
| *Not very interested* | No muy interesado |  | Poco interesado |  |
| *Severe effect* | Efecto severo | Although there was no problem of comprehension and the back-translation was practically the same, it was decided to replace the term "severo" by "grave", since although both terms are correct, in Spanish it is more common within non knowledgeable persons to use the adjective grave in this context | Efecto grave |  |
| *5. On what sort of terrain does your dog most often exercise?*  *On level grass/In woodland/On street/Over rough ground* | 5. ¿Sobre qué tipo de terreno su perro hace ejercicio con más frecuencia?  Sobre césped llano/En el bosque/En la calle/Sobre terreno irregular | One participant suggested adding an additional response option ("Otro"). This was not done because it would mean modifying the original questionnaire, which could have implications for the scoring and interpretation of the results | None | Although there were no comprehension problems, one of the participants in the second cognitive analysis suggested rephrasing the question to "¿Sobre qué tipo de terreno hace ejercicio su perro con más frecuencia?”. This change was not considered as there were no comprehension problems and no further comments |
| *9. What overall effect does exercise have on your dog’s lameness?* | 9. ¿Qué efecto global tiene el ejercicio sobre la cojera de su perro? | One participant suggested rewriting the question: "¿Si realiza más ejercicio de lo habitual, nota que le afecta?" No change was made, as there are no problems with understanding the question, and it would be a relevant change from the original version, which could alter the answers and therefore the total score of the questionnaire | None |  |
| *2. How disabled is your dog by his/her lameness* | 2. ¿Cómo de discapacitado está su perro por la cojera? | One participant suggested changing the question to "¿Qué grado de discapacidad tiene su perro debido a la cojera?". Since there were no problems in reading and understanding the question, and since the Spanish translation matched the original version, no changes were made |  |  |
| *4. What is the effect of cold, damp weather on your dog’s lameness?* | 4. ¿Qué efecto tiene el clima frío y húmedo en la cojera de su perro? | One participant suggested rewriting the question: "¿Cómo afectan el clima frío y húmedo a la cojera de su perro?". Since there were no problems in reading and understanding the question, and since the Spanish translation matched the original version, no changes were made |  |  |
