# Supplementary material for: Translation and linguistic validation into Spanish of the Owner-Reported Outcome Measure “Liverpool Osteoarthritis in Dogs”
Source: Front Vet Sci. 2024 Feb 20;11:1360926. doi: 10.3389/fvets.2024.1360926 (PMC10912287; doi:10.3389/fvets.2024.1360926)
Supplement: Supplementary file 2 [file Data_Sheet_1.DOCX]

Supplementary Material

Translation and linguistic validation into Spanish of the Owner-Reported Outcome Measure “Liverpool Osteoarthritis in Dogs” (LOAD)

**María Olcoz^1†*^, Miguel Ángel Cabezas^2^** **^†^*** **and Ignacio A. Gómez de Segura^1^**

^1^Department of Animal Medicine and Surgery. University Complutense of Madrid. 28040, Madrid, Spain

^2^Dolorvet Anestesia & Analgesia Veterinaria

†These authors contributed equally to this work.

***Correspondence:** Corresponding Author: [mariaolcozcordon@gmail.com](mailto:mariaolcozcordon@gmail.com)

# *Supplementary Data. Questionnaire used in the cognitive debriefing process.*

**Encuesta sobre la legibilidad de la versión española de la escala “Osteoartritis en Perros de Liverpool” de valoración de dolor crónico (LOAD)**

Todavía resulta difícil evaluar el grado de dolor crónico en los perros valorando su comportamiento dada nuestra dificultad para interpretar sus emociones. Para reducir la subjetividad de la valoración del dolor se han diseñado cuestionarios sencillos rellenados por el propietario.

El cuestionario de **Osteoartritis en Perros de Liverpool** (LOAD; en inglés, *Liverpool Osteoarthritis in Dogs*) es un cuestionario de 13 ítems o preguntas para evaluar trastornos articulares en perros como la osteoartritis. La suma de puntuaciones de cada pregunta proporciona una “puntuación LOAD” que sugiere la presencia y gravedad de la enfermedad articular del paciente. Los propietarios completan un cuestionario LOAD inicial en la primera visita y un cuestionario de seguimiento en cada visita posterior.

Sin embargo, un problema frecuente de este tipo de cuestionarios (incluido el **LOAD**) es que han sido desarrollados en inglés y no pueden trasladarse a propietarios de países como España. Para facilitar su uso se ha elaborado una traducción al español, pero debe confirmarse que tiene el mismo significado y las frases son de fácil legibilidad para el propietario medio.

Para verificar dicha legibilidad hemos elaborado tres preguntas sencillas sobre cada pregunta del **LOAD** traducida al español, es decir, si la versión traducida es comprensible para el público en general. Es un formulario voluntario y totalmente anónimo. La información aquí obtenida será utilizada para la elaboración de un Proyecto de Investigación y la implantación de una herramienta que consideramos muy valiosa para mejorar la calidad de vida de los perros.

El formulario se divide en dos partes. La primera, con 3 preguntas de información demográfica básica y anónima. La segunda de valoración de cada una de las preguntas del **LOAD** para saber si se entiende correctamente o genera algún tipo de ambigüedad o dificultad en su lectura y comprensión y, cuando sea oportuno, se proporcione una alternativa que resulte más adecuada.

Si acepta participar en este proyecto y desea rellenar el formulario, por favor marque la casilla correspondiente a continuación:

** Si acepto  No acepto**

Muchas gracias por su colaboración.

El formulario comienza en el reverso de esta hoja

**Marque con una X el rango de edad al que pertenece**

 <29 años  30- 39 años  40-49 años  50-59 años  >60 años

**Marque con una X el género con el que se identifica**

 Hombre  Mujer  Ninguno de los anteriores

**Marque con una X el nivel de estudios**

 Primaria  Secundaria  Bachillerato  Estudios universitarios

_____________________________________________________________________

A continuación, se le van a mostrar uno a uno todos los elementos que componen el **LOAD**, y seguidamente se formularán una serie de preguntas sobre el grado de comprensión de cada elemento:

Estimado propietario,

Gracias por acceder a completar este cuestionario.

Su ayuda en esta tarea nos permitirá recopilar información valiosa sobre su mascota, y es un componente vital en nuestra búsqueda continua para luchar contra enfermedades dolorosas y debilitantes como la artritis. Es importante que responda a todas las preguntas lo mejor que pueda y, si tiene alguna duda sobre el cuestionario, póngase en contacto con un miembro del personal sanitario de su clínica veterinaria.

Gracias de nuevo por su ayuda.

Respondiendo a las preguntas

La mayoría de las preguntas son bastante sencillas. Es importante que marque sólo una casilla por pregunta, salvo que se le pida lo contrario (por ejemplo, la pregunta 4 sobre estilo de vida).

Si tiene alguna duda sobre cómo responder a una pregunta concreta, póngase en contacto con un miembro del personal para que le ayude.

¿Se entiende correctamente el enunciado?

 Si  No

En caso de no comprenderse o haber un término que no se entienda indíquelo a continuación:

En sus propias palabras, ¿redactaría de alguna otra forma el enunciado de modo que le resultara más sencillo de leer o más comprensible? En ese caso indíquelo a continuación:

**Antecedentes**

1. ¿Cuánto tiempo lleva su mascota padeciendo su problema de movilidad?

| Hasta 6 meses | 6 - 12 meses | 12 – 24 meses | 24 – 36 meses | Más de 36 meses |
| --- | --- | --- | --- | --- |

¿Se entiende correctamente la pregunta, así como las posibles opciones de respuesta?

 Si  No

En caso de no comprenderse la pregunta o haber un término que no se entienda indíquelo a continuación:

En sus propias palabras, ¿redactaría de alguna otra forma la pregunta de modo que le resultara más sencillo de leer o más comprensible? En ese caso indíquelo a continuación:

1. ¿Se le ha diagnosticado a su perro, o padece, algún otro problema además de su enfermedad ortopédica?

| No | Si | Enumérelos si puede: |
| --- | --- | --- |

¿Se entiende correctamente la pregunta, así como las posibles opciones de respuesta?

 Si  No

En caso de no comprenderse la pregunta o haber un término que no se entienda indíquelo a continuación:

En sus propias palabras, ¿redactaría de alguna otra forma la pregunta de modo que le resultara más sencillo de leer o más comprensible? En ese caso indíquelo a continuación:

1. Si puede, por favor haga una lista de los medicamentos que esté tomando su mascota, indicando cuándo recibió la última dosis de cada uno:

¿Se entiende correctamente la pregunta, así como las posibles opciones de respuesta?

 Si  No

En caso de no comprenderse la pregunta o haber un término que no se entienda indíquelo a continuación:

En sus propias palabras, ¿redactaría de alguna otra forma la pregunta de modo que le resultara más sencillo de leer o más comprensible? En ese caso indíquelo a continuación:

**Estilo de vida**

1. En la última semana, de promedio ¿qué distancia ha recorrido su perro cada día?

| 0 – 1 km | 1 – 2 km | 2 – 3 km | 3 – 4 km | Más de 4 km |
| --- | --- | --- | --- | --- |

¿Se entiende correctamente la pregunta, así como las posibles opciones de respuesta?

 Si  No

En caso de no comprenderse la pregunta o haber un término que no se entienda indíquelo a continuación:

En sus propias palabras, ¿redactaría de alguna otra forma la pregunta de modo que le resultara más sencillo de leer o más comprensible? En ese caso indíquelo a continuación:

1. En la última semana, de promedio ¿cuántos paseos ha dado su perro al día?

| 0 | 1 | 2 | 3 | 4 | Más de 4 |
| --- | --- | --- | --- | --- | --- |

¿Se entiende correctamente la pregunta, así como las posibles opciones de respuesta?

 Si  No

En caso de no comprenderse la pregunta o haber un término que no se entienda indíquelo a continuación:

En sus propias palabras, ¿redactaría de alguna otra forma la pregunta de modo que le resultara más sencillo de leer o más comprensible? En ese caso indíquelo a continuación:

1. ¿Cómo hace esta actividad?

| Siempre  con correa | Casi siempre con correa | Casi siempre  sin correa | Siempre  sin correa | Trabajando  (perro de trabajo) |
| --- | --- | --- | --- | --- |

¿Se entiende correctamente la pregunta, así como las posibles opciones de respuesta?

 Si  No

En caso de no comprenderse la pregunta o haber un término que no se entienda indíquelo a continuación:

En sus propias palabras, ¿redactaría de alguna otra forma la pregunta de modo que le resultara más sencillo de leer o más comprensible? En ese caso indíquelo a continuación:

1. ¿Hay días concretos de la semana en los que su perro hace bastante más ejercicio? (Marque más de una casilla si es necesario)

| Lunes | Martes | Miércoles | Jueves | Viernes | Sábado | Domingo |
| --- | --- | --- | --- | --- | --- | --- |

¿Se entiende correctamente la pregunta, así como las posibles opciones de respuesta?

 Si  No

En caso de no comprenderse la pregunta o haber un término que no se entienda indíquelo a continuación:

En sus propias palabras, ¿redactaría de alguna otra forma la pregunta de modo que le resultara más sencillo de leer o más comprensible? En ese caso indíquelo a continuación:

1. ¿Sobre qué tipo de terreno su perro hace ejercicio con más frecuencia?

| Sobre césped llano | Sobre terreno boscoso | En la calle | Sobre terreno accidentado |
| --- | --- | --- | --- |

¿Se entiende correctamente la pregunta, así como las posibles opciones de respuesta?

 Si  No

En caso de no comprenderse la pregunta o haber un término que no se entienda indíquelo a continuación:

En sus propias palabras, ¿redactaría de alguna otra forma la pregunta de modo que le resultara más sencillo de leer o más comprensible? En ese caso indíquelo a continuación:

1. Cuando hace ejercicio, ¿cómo va a su perro?

| Camina con correa | Camina sin correa | Trota | Corre libremente |
| --- | --- | --- | --- |

¿Se entiende correctamente la pregunta, así como las posibles opciones de respuesta?

 Si  No

En caso de no comprenderse la pregunta o haber un término que no se entienda indíquelo a continuación:

En sus propias palabras, ¿redactaría de alguna otra forma la pregunta de modo que le resultara más sencillo de leer o más comprensible? En ese caso indíquelo a continuación:

1. ¿Quién limita cuánto ejercicio hace su perro?

| Usted | Su perro |
| --- | --- |

¿Se entiende correctamente la pregunta, así como las posibles opciones de respuesta?

 Si  No

En caso de no comprenderse la pregunta o haber un término que no se entienda indíquelo a continuación:

En sus propias palabras, ¿redactaría de alguna otra forma la pregunta de modo que le resultara más sencillo de leer o más comprensible? En ese caso indíquelo a continuación:

**Movilidad**

**Generalmente**

1. ¿Cómo es la movilidad de su perro en general?

| Muy buena | Buena | Aceptable | Mala | Muy mala |
| --- | --- | --- | --- | --- |

¿Se entiende correctamente la pregunta, así como las posibles opciones de respuesta?

 Si  No

En caso de no comprenderse la pregunta o haber un término que no se entienda indíquelo a continuación:

En sus propias palabras, ¿redactaría de alguna otra forma la pregunta de modo que le resultara más sencillo de leer o más comprensible? En ese caso indíquelo a continuación:

1. ¿Cómo de discapacitado está su perro por la cojera?

| Ninguna discapacidad | Levemente discapacitado | Moderadamente discapacitado | Gravemente discapacitado | Extremadamente discapacitado |
| --- | --- | --- | --- | --- |

¿Se entiende correctamente la pregunta, así como las posibles opciones de respuesta?

 Si  No

En caso de no comprenderse la pregunta o haber un término que no se entienda indíquelo a continuación:

En sus propias palabras, ¿redactaría de alguna otra forma la pregunta de modo que le resultara más sencillo de leer o más comprensible? En ese caso indíquelo a continuación:

1. ¿Cómo de activo es su perro?

| Extremadamente activo | Muy  activo | Moderadamente activo | Levemente activo | Nada  activo |
| --- | --- | --- | --- | --- |

¿Se entiende correctamente la pregunta, así como las posibles opciones de respuesta?

 Si  No

En caso de no comprenderse la pregunta o haber un término que no se entienda indíquelo a continuación:

En sus propias palabras, ¿redactaría de alguna otra forma la pregunta de modo que le resultara más sencillo de leer o más comprensible? En ese caso indíquelo a continuación:

1. ¿Qué efecto tiene el clima frío y húmedo en la cojera de su perro?

| Sin efecto | Efecto leve | Efecto moderado | Efecto grave | Efecto extremo |
| --- | --- | --- | --- | --- |

¿Se entiende correctamente la pregunta, así como las posibles opciones de respuesta?

 Si  No

En caso de no comprenderse la pregunta o haber un término que no se entienda indíquelo a continuación:

En sus propias palabras, ¿redactaría de alguna otra forma la pregunta de modo que le resultara más sencillo de leer o más comprensible? En ese caso indíquelo a continuación:

1. ¿Hasta qué grado su perro muestra rigidez en la extremidad afectada después de estar tumbado?

| Sin rigidez | Rigidez leve | Rigidez moderada | Rigidez grave | Rigidez extrema |
| --- | --- | --- | --- | --- |

¿Se entiende correctamente la pregunta, así como las posibles opciones de respuesta?

 Si  No

En caso de no comprenderse la pregunta o haber un término que no se entienda indíquelo a continuación:

En sus propias palabras, ¿redactaría de alguna otra forma la pregunta de modo que le resultara más sencillo de leer o más comprensible? En ese caso indíquelo a continuación:

**Durante el ejercicio**

1. Cuando hace ejercicio, ¿cómo de activo es su perro?

| Extremadamente activo | Muy  activo | Aceptablemente activo | Poco  activo | Nada  activo |
| --- | --- | --- | --- | --- |

¿Se entiende correctamente la pregunta, así como las posibles opciones de respuesta?

 Si  No

En caso de no comprenderse la pregunta o haber un término que no se entienda indíquelo a continuación:

En sus propias palabras, ¿redactaría de alguna otra forma la pregunta de modo que le resultara más sencillo de leer o más comprensible? En ese caso indíquelo a continuación:

1. ¿Qué interés tiene su perro en hacer ejercicio?

| Extremadamente interesado | Muy  interesado | Aceptablemente interesado | Poco interesado | Nada  interesado |
| --- | --- | --- | --- | --- |

¿Se entiende correctamente la pregunta, así como las posibles opciones de respuesta?

 Si  No

En caso de no comprenderse la pregunta o haber un término que no se entienda indíquelo a continuación:

En sus propias palabras, ¿redactaría de alguna otra forma la pregunta de modo que le resultara más sencillo de leer o más comprensible? En ese caso indíquelo a continuación:

1. ¿Cómo calificaría la capacidad de su perro para hacer ejercicio?

| Muy buena | Buena | Aceptable | Mala | Muy mala |
| --- | --- | --- | --- | --- |

¿Se entiende correctamente la pregunta, así como las posibles opciones de respuesta?

 Si  No

En caso de no comprenderse la pregunta o haber un término que no se entienda indíquelo a continuación:

En sus propias palabras, ¿redactaría de alguna otra forma la pregunta de modo que le resultara más sencillo de leer o más comprensible? En ese caso indíquelo a continuación:

1. ¿Qué efecto global tiene el ejercicio sobre la cojera de su perro?

| Sin efecto | Efecto leve | Efecto moderado | Efecto grave | Efecto extremo |
| --- | --- | --- | --- | --- |

¿Se entiende correctamente la pregunta, así como las posibles opciones de respuesta?

 Si  No

En caso de no comprenderse la pregunta o haber un término que no se entienda indíquelo a continuación:

En sus propias palabras, ¿redactaría de alguna otra forma la pregunta de modo que le resultara más sencillo de leer o más comprensible? En ese caso indíquelo a continuación:

1. ¿Con qué frecuencia descansa su perro (se para/se sienta) durante el ejercicio?

| Nunca | Casi nunca | Ocasionalmente | Frecuentemente | Muy frecuentemente |
| --- | --- | --- | --- | --- |

¿Se entiende correctamente la pregunta, así como las posibles opciones de respuesta?

 Si  No

En caso de no comprenderse la pregunta o haber un término que no se entienda indíquelo a continuación:

En sus propias palabras, ¿redactaría de alguna otra forma la pregunta de modo que le resultara más sencillo de leer o más comprensible? En ese caso indíquelo a continuación:

1. ¿Qué efecto tiene el clima frío y húmedo en la capacidad de su mascota para hacer ejercicio?

| Sin efecto | Efecto leve | Efecto moderado | Efecto grave | Efecto extremo |
| --- | --- | --- | --- | --- |

¿Se entiende correctamente la pregunta, así como las posibles opciones de respuesta?

 Si  No

En caso de no comprenderse la pregunta o haber un término que no se entienda indíquelo a continuación:

En sus propias palabras, ¿redactaría de alguna otra forma la pregunta de modo que le resultara más sencillo de leer o más comprensible? En ese caso indíquelo a continuación:

1. ¿Hasta qué grado su perro muestra rigidez en la extremidad afectada después de haber estado tumbado tras el ejercicio?

| Sin rigidez | Rigidez leve | Rigidez moderada | Rigidez grave | Rigidez extrema |
| --- | --- | --- | --- | --- |

¿Se entiende correctamente la pregunta, así como las posibles opciones de respuesta?

 Si  No

En caso de no comprenderse la pregunta o haber un término que no se entienda indíquelo a continuación:

En sus propias palabras, ¿redactaría de alguna otra forma la pregunta de modo que le resultara más sencillo de leer o más comprensible? En ese caso indíquelo a continuación:

1. ¿Qué efecto tiene la cojera de su perro en su capacidad para hacer ejercicio?

| Sin efecto | Efecto leve | Efecto moderado | Efecto grave | Efecto extremo |
| --- | --- | --- | --- | --- |

Gracias una vez más por completar este cuestionario.

Por favor, devuelva el formulario a un miembro del personal.

¿Se entiende correctamente la pregunta, así como las posibles opciones de respuesta?

 Si  No

En caso de no comprenderse la pregunta o haber un término que no se entienda indíquelo a continuación:

En sus propias palabras, ¿redactaría de alguna otra forma la pregunta de modo que le resultara más sencillo de leer o más comprensible? En ese caso indíquelo a continuación:
